# Automated Methodology for Dependability Evaluation of Wireless Visual Sensor Networks

**DOI:** 10.3390/s18082629

**Published:** 2018-08-10

**Authors:** Thiago C. Jesus, Paulo Portugal, Francisco Vasques, Daniel G. Costa

**Affiliations:** 1 INEGI/INESC-TEC—Faculty of Engineering, University of Porto (FEUP), 4200-465 Porto, Portugal; pportugal@fe.up.pt (P.P.); vasques@fe.up.pt (F.V.); 2 Department of Technology, State University of Feira de Santana (DTEC/UEFS), 44036-900 Feira de Santana, Brazil; danielgcosta@uefs.br

**Keywords:** dependability evaluation, availability, reliability, wireless visual sensor networks, fault tree analysis, Markov chains

## Abstract

Wireless sensor networks have been considered as an effective solution to a wide range of applications due to their prominent characteristics concerning information retrieving and distributed processing. When visual information can be also retrieved by sensor nodes, applications acquire a more comprehensive perception of monitored environments, fostering the creation of wireless visual sensor networks. As such networks are being more often considered for critical monitoring and control applications, usually related to catastrophic situation prevention, security enhancement and crises management, fault tolerance becomes a major expected service for visual sensor networks. A way to address this issue is to evaluate the system dependability through quantitative attributes (e.g., reliability and availability), which require a proper modeling strategy to describe the system behavior. That way, in this paper, we propose a methodology to model and evaluate the dependability of wireless visual sensor networks using Fault Tree Analysis and Markov Chains. The proposed modeling strategy considers hardware, battery, link and coverage failures, besides considering routing protocols on the network communication behavior. The methodology is automated by a framework developed and integrated with the SHARPE (Symbolic Hierarchical Automated Reliability and Performance Evaluator) tool. The achieved results show that this methodology is useful to compare different network implementations and the corresponding dependability, enabling the uncovering of potentially weak points in the network behavior.

## 1. Introduction

Wireless Sensor Networks (WSNs) are nowadays a trend as a support technology for many types of monitoring applications. Recently, applications in the field of smart cities, street lighting, domotics, traffic and pedestrian control, living assistance, parking assistance, waste collection and surveillance activities are becoming common, since they present data volumes and computational demands perfectly suitable for WSNs. Some of these applications may require the addition of visual information to the data set [1]. For example, a street lighting application triggered by motion sensors can be wrongly activated upon the presence of birds; a traffic light control system can better manage its time intervals with an image of how many people or cars are occupying the sidewalk or the road; an intelligent transportation system can easily use images to detect a car accident and then call the proper authorities in addition to the obvious usage of visual data, like surveillance, face detection, intrusion detection, etc. All of these problems can be supported by the use of adequate Wireless Visual Sensor Networks (WVSNs) [2,3,4].

Many applications in different monitoring and control domains are said to be safety related. In such kind of systems, a system failure may put people in danger, lead to environmental damages or result in economic losses. Therefore, it is mandatory to evaluate the system dependability in those situations in order to assess its successful operational behavior through the time, although there are relevant challenges to be handled for such assessment.

Actually, dependability is a generic concept including attributes as availability, reliability, safety, integrity and maintainability [5], being commonly addressed by its quantitative attributes (reliability and availability) [6,7,8,9]. According to Avizienis et al. [5], reliability can be defined as the probability of a system to perform its purpose adequately (failure-free) for the intended period of time, under the operating conditions found in a specified environment. On the other hand, availability is the probability of finding the system in a correct operating state at some time into the future, or, in another way, it is the amount of time that a system is actually operating, as the percentage of the total time that it should be operating. One should note that reliability is associated with the system behavior until a failure occurs, while availability is associated with the system behavior even if failures occur. In this case, repair actions are required to take the system from a failed state to an operational state.

In order to evaluate dependability, different approaches can be used, commonly categorized as *analytical*, *simulation* and *experimental*, each one presenting a different level of accuracy, cost, duration and comprehensiveness. Some authors also define the *formal* category [10], but it is assumed herein that it is equivalent to the analytical approach, since the tools to model systems in a formal way can also be described analytically. While experimental approaches require the existence of a real system to perform the evaluation at run-time, analytical and simulation approaches are easier to implement because they are based on models. However, the accuracy of their results depend on the accuracy of the values assigned to the model parameters and the considered hypothesis. Moreover, the use of simulation for dependability evaluation brings an important concern: for an accurate estimation of dependability measures, frequent observations of the system-failure event are necessary, which, by definition are rare events. This results in a substantial increase of the simulation time, which could lead to impractical values [8,10,11,12]. Therefore, considering this complex scenario for dependability evaluation, in this paper, we will focus on analytical modeling, since it is generally cheaper, faster and can provide an acceptable level of accuracy.

In this paper, we propose a methodology to evaluate the dependability of Wireless Visual Sensor Networks. This methodology is based on an analytical approach, using Fault Tree Analysis (FTA) and Continuous Time Markov Chains (CTMCs) to model the network behavior, considering battery, communication links, hardware and visual coverage failures, and the impact of different routing strategies on WVSNs. Since the analytical modeling can be a time-consuming task, the entire methodology has been automated by a framework integrated with the SHARPE (Symbolic Hierarchical Automated Reliability and Performance Evaluator) tool [13], which is used to solve the models numerically.

The proposed methodology significantly improves both the methodologies proposed by Silva et al. [6] to evaluate the reliability and availability of WSNs in industrial environments and by Costa et al. [14] to evaluate availability of WVSNs for target coverage. We improve these methodologies to support an integrated evaluation of WVSNs’ dependability, adapted to the context of area coverage monitoring, in a way that it is possible to specify network failure conditions as a function of a coverage objective. The dependability metrics proposed are mainly focused on the availability. However, it is important to notice that they can be easily adaptable to evaluate the reliability, as shown later.

The remainder of this paper is organized as follows: Section 2 presents related works regarding dependability on WSNs and WVSNs. In Section 3, the problem formulation is stated and the theoretical background is presented. The proposed methodology is described in Section 4, detailing the modeling and evaluation phases. Section 5 presents and discusses the results according to the evaluation of different WVSN scenarios. Finally, conclusions are stated in Section 6.

## 2. Related Works

Due to its importance, dependability evaluation is an issue widely addressed in the literature, especially on WSNs. Several papers can be founded surveying some aspect of dependability on WSNs [10,15,16,17,18,19,20,21,22,23,24,25], while some others papers survey WVSNs [26,27,28,29,30,31] as their main research areas. However, just a few works have discussed dependability aspects of WVSNs, with emphasis on the availability assessment [14,32,33,34,35].

Bruneo et al. [36] analytically evaluates dependability issues in terms of reliability and *producibility* (a new attribute introduced in that work), modeling the behavior of sensor nodes using Markov Reward models. The energy consumption is computed considering that a sensor can be in active or sleep state, and the battery charge is estimated in order to detect a failure.  his information is used to evaluate reliability and *producibility* of each sensor node, and an algorithm is proposed to evaluate WSNs’ dependability, taking into account the network topology and the sensor redundancy.

New concepts related to dependability are also proposed by Huang et al. [37]. In that paper, an approach is presented to quantify reliability and availability, in addition to maintainability, safety and integrity. These dependability attributes are evaluated based on metrics such as mean time to reliability failure (MTTRF), mean time to availability failure (MTTAF), mean time to maintainability failure (MTTMF), mean time to safety failure (MTTSF) and mean time to integrity failure (MTTIF).

Maza [38] uses Stochastic Activity Networks (SAN) to model time-continuous systems, including maintenance and fault diagnosis aspects. Moreover, through Monte Carlo Simulation, the authors also assess dependability attributes such reliability and availability. For this purpose, it is shown how to model sensors faults, measurement noise, and how to use these data to evaluate dependability.

Macedo et al. [39] consider the Internet of Things context to evaluate dependability, although, according to the authors, their approach can be adapted to evaluate more complex scenarios, assuming any routing strategy and topology types. Systems are modeled by Markov Chains, which are evaluated using the SHARPE tool. These models consider node redundancy and the mean time to failure (MTTF) of the system, metrics that can be used to assess reliability and availability.

Since analytical modeling is a time-consuming task, demanding much effort for complex topologies, some authors propose automated model generation approaches [6,8,12,40,41]. Cinque et al. [12] propose a framework to assess both dependability and performance of WSNs through automatic generation of analytical models. The proposed modeling approach considers unreliable devices and unreliable links, power consumption, routing protocols, workload and radio specifications. The proposed framework integrates behavioral models (analyzed by the AVRORA simulator [42]) and analytical models (described by SAN formalism). In addition, the proposed framework is capable of inferring a realistic WSN model and to evaluate this model with respect to connection resiliency, coverage level of the monitored area, data delivery resiliency and efficiency, availability, lifetime and isolation time of nodes. Nevertheless, the model templates must be predefined *una-tantum* by a domain expert.

Silva et al. [6] propose a methodology for automatic generation of analytical dependability models based on FTA, in industrial environments that are subject to permanent faults on network devices. The methodology is integrated with the SHARPE tool and evaluates the application behavior with regard to reliability, unreliability, availability, unavailability, MTTF and component importance (Birnbaum and Criticality), considering line, star, cluster and mesh topologies.

Dâmaso et al. [40] present a modeling strategy to evaluate the reliability of WSNs, considering the battery level as a key factor, but also considering routing algorithms, unreliable devices and unreliable links. The WSN power consumption is evaluated by Coloured Petri Net (CPN) models that are composed by basic models, which represent the power consumption of small parts of the application or the network. A tool is also proposed to automate the evaluation of WSNs reliability. This work has been extended in [41], in order to support analyses with the power consumption of WSN applications and communication protocols.

Martins et al. [8] propose a toolset to support the evaluation of the dependability of WSNs in industrial environments, focusing on the automatic generation of analytical dependability models from AADL (Architecture Analysis and Design Language) architecture models. The proposed framework is integrated with the SHARPE tool and decides which modeling technique is the most appropriate for each case according to the system structure and dependability metrics.

Regarding WVSNs,  [32] identify and discuss the main availability issues of this application domain. This paper focuses on how redundancy should be considered to improve the availability level of WVSNs, with respect to a camera’s field of view (FoV) overlapping, sensing similarity and sensing relevance. Moreover, the authors discuss common hardware and coverage failures [43] that can affect such availability level. The authors indicate that the availability evaluation in WVSNs has to consider coverage quality, quality of viewing, barrier monitoring, directional k-coverage and users’ perceptions. In [32], practical approaches and mechanisms to evaluate and to enhance availability in WVSNs are also discussed, and, in [44], authors discuss routing mechanisms based on the sensing relevancies of source nodes for critical applications in WVSNs.

Redundancy in WVSNs is considered in [33,35], and used as a dependability metric. In these cases, availability is evaluated with respect to the redundancy level. The authors compute the FoV of each visual sensor node in order to know the application FoV and to select the redundant nodes. In [33], this metric is evaluated considering the minimum percentage of FoV and the maximum acceptable angle between sensors’ orientation. In [35], occlusion is added as a redundancy parameter, changing the way to compute FoV. Additionally, an algorithm to adjust cameras’ orientations is proposed to enhance the availability of WVSNs with occlusion. In [45], the selection of sensor nodes for barrier coverage monitoring is also addressed based on redundancy coverage level of the nodes.

In [34], a new coverage metric is proposed, the Effective Target Viewing (ETV), which characterizes the percentage of viewed parts of targets’ perimeters. This metric is exploited to assess the availability of WVSN monitoring applications. In this case, ETV is associated with an availability state, which may be “yes” (available) or “no” (unavailable), according to the defined Minimum acceptable ETV (M-ETV) threshold. However, although addressing dependability issues of WVSNs, the papers [33,34,35] only address coverage aspects, ignoring communication and hardware issues.

Costa et al. [14] partially address these issues using the methodology proposed by Silva et al. [6] to perform availability assessments in WVSNs. This evaluation is associated with all visual sensors nodes that are monitoring a target, considering hardware failures (battery discharging), communication failures (loss of path to a sink node) and coverage failures (loss of view over targets). However, communication failures are modeled in such a way that did not consider the effects of routing protocols. The network failure condition (NFC) is identified by a voting gate (*k-out-of-N*), meaning that whenever *k* out of the *N* visual sensors nodes monitoring the target fail, the application will fail, ignoring the different importance degree of sensor nodes.

In order to model routing protocols and to integrate them into the dependability models, Kafi et al. [46] surveys the reliable routing protocols on WSNs and, more specifically, Hasan et al. [31] surveys’ multipath routing protocols on Wireless Multimedia Sensor Networks (WMSNs), which is a generalization of WVSNs. In [47,48], routing protocols are addressed considering energy consumption in order to preserve the network reliability. Those papers present relevant features from several routing protocols classified by suitable application scenarios, energy efficiency and real-time aspects, providing a basis to categorize them and model their behavior with respect to dependability. Additionally, Zonouz et al. [49] present wireless link reliability models for energy harvesting and battery-powered sensor nodes, considering power consumption, noise, location uncertainty and wireless channel conditions.

Differently from previous papers, in this work, we propose a methodology to analytically evaluate dependability metrics of wireless visual sensor networks, considering essential aspects in an integrated fashion, i.e., the influence of power consumption, battery discharging, hardware, link and coverage failures, as well as the behavior of routing protocols. To the best of our knowledge, our framework is the only one in the literature that puts together all of these features, providing an automated model generation approach.

## 3. Problem Formulation and Background

One of the main goals of this paper is to develop an integrated analytical model that reflects the characteristics of the whole application and that allows for computing its dependability. This model is usually an abstraction of the system behavior, being composed by a network of components characterized by their failure distributions and maintenance policies [50].

System dependability, roughly speaking, can be seen as a service that may or may not be correctly provided at a given instant of time, usually associated to the achievement of some metric related to the application. In this work, the imposed requirement is that the WVSN application needs to monitor a minimum percentage of the area of interest. The achievement of this goal depends on the components that are operating at any instant and the interaction among them.

WVSN components are basically its nodes. Similarly to Costa et al. [14], in this paper, we assume three types of nodes: sink, visual and scalar. The sink node is a base station, the common destination of all information collected by nodes in the network. Visual nodes are sensor nodes equipped with cameras that gather images from the monitored area. Scalar nodes are sensor nodes unable to collect visual information, but able to re-transmit it, working as relaying nodes.

A node can fail if at least one of its hardware components fails, i.e., processor, memory, radio, battery and sensor units (e.g., camera). However, for modelling purposes, we partitioned node components in two categories: battery and generic hardware (processor, memory, radio and sensors). This partition is made because these categories present different behaviors, so we propose different models for them. We consider that a node failure is permanent, requiring a replacement or repair action to come back to the operational state. A hardware failure can be a broken electronic component, while we consider a battery failure as equivalent to its full discharge. A hardware repair can be a component replacement or a component fixing. A battery repair can be a replacement or a recharging.

Nodes interact between them through message exchanges. This interaction can fail if, obviously, nodes fail or if the communication links fail. We consider that a link failure is transient, meaning that the failed link will reestablish its connection after a while, without an external intervention. A link failure could be a radio interference, an occupied channel or a data collision. Once a communication failure occurs, the connection from a node to the sink node could be reestablished by the re-routing or self-healing features of routing protocols.

The availability evaluation of a WVSN is focused on area coverage and involves all the previous network elements. If the hardware of a visual node fails, it will be incapable of collecting visual information. On the other hand, if a link or an intermediate node that composes a path to the sink node fails, then the visual information of one or more nodes will not be delivered to the sink node. In any case, failures will not allow the sink node to receive all of the visual information related to that minimum percentage. Thus, the WVSN application requirements are not achieved and a visual coverage failure (VCF, hereafter referred as coverage failure) occurs. That way, in order to evaluate the availability of a WVSN, it is necessary to define and to model each element that can fail, besides the relationships among them.

The next subsections present the proposed dependability models for nodes and links, as well as components directly related to them. First, it will be shown how to compute the coverage area, which allows for identifying the occurrence of a coverage failure, and finally it will be shown how to integrate all those models in the evaluation process.

### 3.1. Area Coverage

The formalism of how to compute the total area viewed by visual nodes is discussed in this section.

**Figure 1:**
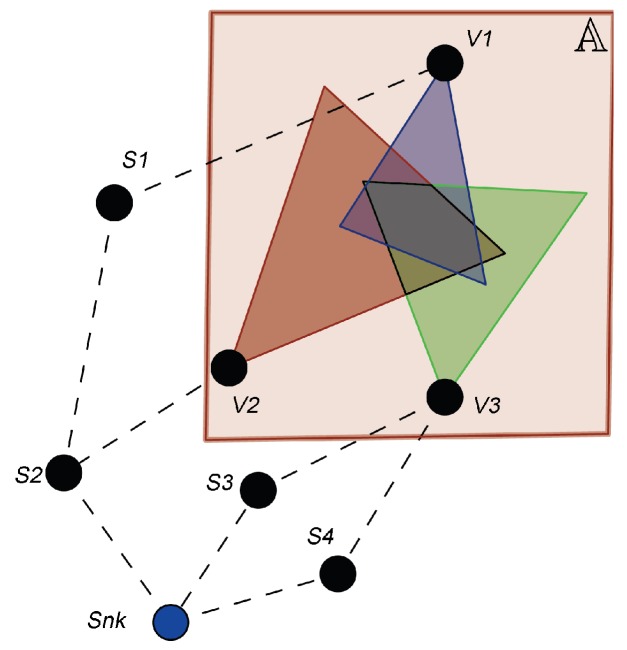
Area monitoring by visual sensors [14].

Consider a wireless visual sensor network being defined as $wvsn=\left\{\left\{Snk\right\}\cup VS\cup SS\right\}$, where Snk is the sink node (base station), $VS=\left\{vs_{i}|i=1,\ldots ,n\right\}$ is a set of visual sensor nodes and $SS=\left\{ss_{j}|j=1,\ldots ,m\right\}$ is a set of scalar sensor nodes. Visual nodes are responsible for monitoring the area of interest A, while scalar nodes can be used as routers (relaying nodes) to support the delivery of visual information to the sink node [14]. Figure 1 shows a WVSN with visual nodes V1, V2 and V3 that can reach the sink by assistance of the scalar nodes S1, S2, S3 and S4.

**Figure 2:**
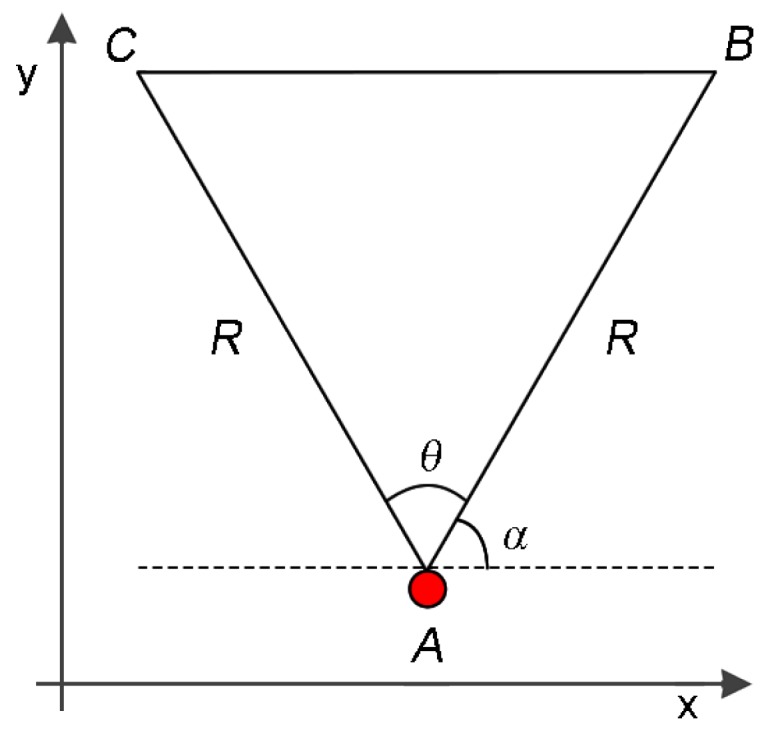
Field of View (FoV) of a visual sensor [34].

In a typical WVSN, each visual node has an embedded camera with a viewing angle θ and an orientation α (see Figure 2). The camera also defines a sensing radius *R* that is an approximation of the camera’s Depth of Field (DoF) [34,51]. For simplification, the Field of View (FoV) of any visual sensor is defined as the area of an isosceles triangle composed of three vertices, *A*, *B* and *C*, being $\left(A_{x},A_{y}\right)$ the Cartesian coordinates of the camera. The coordinates of vertices *B* and *C* can be obtained by Equation (1) and the FoV of any visual sensor vs (${\mathrm{FoV}}_{vs}$) can be computed using trigonometry, as expressed in Equation (2) [14,34]:

(1) $$\begin{array}{c} B_{x}=A_{x}+R\cos\left(\alpha \right),\\ B_{y}=A_{y}+R\sin\left(\alpha \right),\\ C_{x}=A_{x}+R\cos\left(\left(\alpha +\theta \right)\;\mathrm{mod}\;2\pi \right),\\ C_{y}=A_{y}+R\sin\left(\left(\alpha +\theta \right)\;\mathrm{mod}\;2\pi \right), \end{array}$$

(2) $${\mathrm{FoV}}_{vs}=\frac{R_{vs}^{2}\cdot \sin\left(\theta_{vs}\right)}{2}.$$

**Figure 3:**
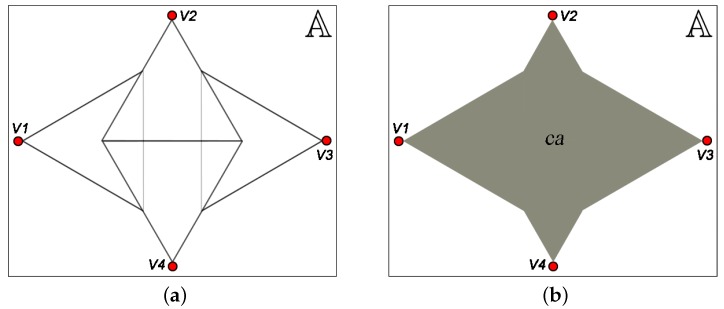
(**a**) monitored area A and (**b**) the coverage area $ca=CA\left(\left\{1,2,3,4\right\}\right)$.

Figure 3a shows the monitored area A and the FoV of four visual nodes. The coverage area ca is the sum of all FoV, considering properly the overlapped area, as shown in Figure 3b. In this case, the coverage area can be computed according to the Inclusion–Exclusion Principle, which is a counting technique from combinatorial mathematics. That principle computes the number of objects in a union of sets, for the most general of circumstances in which the sets are free to overlap without restriction [52,53]. It is stated in Theorem 1, and so the coverage area can be computed according to Definition 1.

**Theorem** **1** (Inclusion–Exclusion Principle). *Suppose n∈N and $A_{i}$ is a finite set for 1≤i≤n. It follows that [52,53,54]*

(3) $$\left|\overset{n}{\underset{i=1}{⋃}}A_{i}\right|=\sum_{\emptyset \neq Q\subseteq \left\{1,2,\ldots ,n\right\}}{\left(-1\right)}^{\left|Q\right|-1}\cdot \left|\underset{q\in Q}{⋂}A_{q}\right|.$$

**Proof.**  See [52]. ☐

Considering a covered region A as a set of points and the cardinality of that set as the area of that region, then the Inclusion–Exclusion Principle can be adjusted and the resulting coverage area can be computed according to Definition 1.

**Definition** **1** (Coverage Area). *Let A be a monitoring area, $VS=\left\{vs_{i}|i=1,\ldots ,n\right\}$ a set of visual nodes covering A and Area(p) the area of the polygon p, which defines a covered region. The coverage area of VS is defined as:*

(4) $$CA\left(VS\right)=\sum_{\emptyset \neq Q\subseteq VS}{\left(-1\right)}^{\left|Q\right|-1}\cdot Area\left(\underset{vs_{q}\in Q}{⋂}vs_{q}\cap A\right).$$

As mentioned before, the coverage area computed by Definition 1 is used as metric to identify whether a coverage failure occurred. Thus, this metric will help to determine which network elements cannot fail so that the application still meets its requirements.

### 3.2. Node Modeling

In this section, the dependability model for sensors nodes is presented. As stated previously, we split the proposed model in two parts: the (generic) hardware model and the battery model, presented as follows.

#### 3.2.1. Hardware Modeling

We consider that the hardware of the node is composed of the following electronic components: processor, memory, radio and sensing unit (camera). For these elements, it is assumed that a failure is permanent, which means that a component replacement or a repair action is required to return the component to an operational state. It is also considered that hardware failures have a random nature and occur according to a Poisson process, i.e., with a constant rate during the useful life period of the node [55].

Regarding the repair processes, we assume that the repair time can be approximated by an exponential distribution (i.e., a constant repair rate). This kind of approximation is reasonable when failure and repair rates differ from each other by several orders of magnitude. We also assume that a repair action repairs all faulty components and that the number of repair actions is not bounded [56]. Moreover, failure and repair actions are assumed i.i.d. (independent and identically distributed) random variables [6]. That way, it is possible to summarize the hardware failure rate of a node as a single and constant failure rate $\lambda_{hw}$, resulting in the sum of failure rates of each component. In an analogous way, hardware repair actions of a node can be summarized by a single and constant repair rate $\mu_{hw}$.

**Figure 4:**
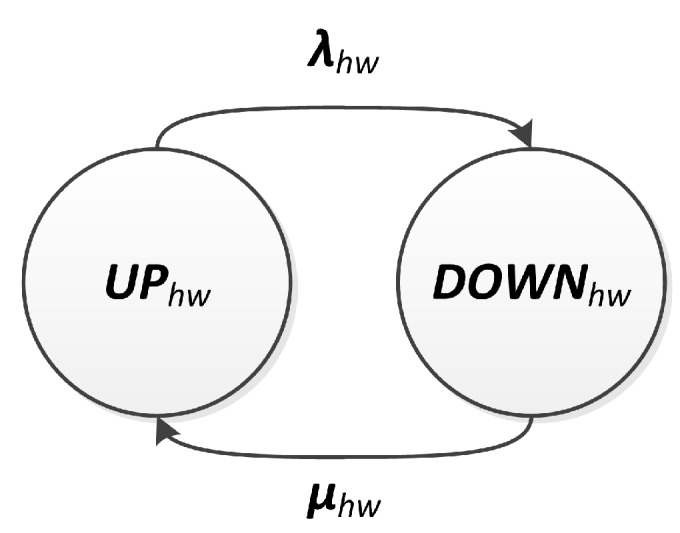
Hardware model.

The hardware behavior with respect to availability is described as a binary relationship, which can be operable (UP) or failed (DOWN). In the former case, the components are operational, and, in the latter, they are failed. This behavior can be represented by a CTMC with two states ($UP_{hw}$) and ($DOWN_{hw}$). Transitions between these two CTMCs’ states are described by the failure and repair rates, $\lambda_{hw}$ and $\mu_{hw}$, respectively. Figure 4 shows the proposed hardware model. Under the stated assumptions, the hardware availability, $hw=A_{hw}\left(t\right)$, can be computed as the probability of being on state $UP_{hw}$.

### 3.3. Battery Modeling

Based on Peukert’s law [57,58,59], which expresses the battery lifetime given an initial capacity *C*, the trend of the battery discharging process with respect to the time is represented by Equation (5). In this equation, $c_{0}$ is the initial capacity of the battery (expressed in Ampere·hour), *I* is the average continuous discharge current (measured in Ampere), *H* is the hour rating (hours), whereas η expresses the Peukert’s constant, which depends on the battery material (e.g., 1.06 to 1.13 for lithium ion batteries and 1.2 to 1.4 for alkaline batteries).

(5) $$c\left(t\right)=c_{0}-I\cdot H\cdot {\left(\frac{t}{H}\right)}^{\frac{1}{\eta }}.$$

The battery behavior presented in Equation (5) is nonlinear and cannot be modeled using the same reasoning of the hardware modeling. To cope with this problem and to evaluate the battery availability, we propose an approximation of the nonlinear battery discharging behavior by a stochastic process, following the approach proposed by Bruneo et al. [60].

**Figure 5:**
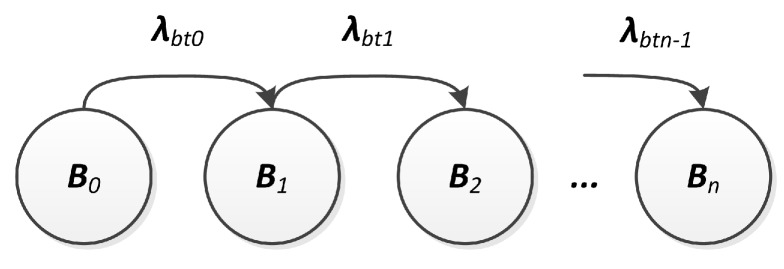
Battery discharging model.

First, the battery’s useful charge range is identified as $\left[c_{0},c_{\min}\right]$, splitting it into *n* contiguous intervals $\left[c_{i},c_{i+1}\right]$ of equal size c0-cminc0-cminnn, with i=0,⋯,n-1. That way, the battery capacity is discretized into n+1 charge levels with generic value $c_{i}=c\left(t_{i}\right)$ (i=0,⋯,n), where $c_{n}=c_{\min}$. It is assumed that the duration of the *i*-th time interval, $\tau_{i}=t_{i+1}-t_{i}$, with i=0,⋯,n-1, can be described by an exponential distribution, in which the charge assumes values ranging into $\left[c_{i},c_{i+1}\right]$. Based on these assumptions, the discharge phenomenon can be represented by a CTMC with n+1 stages, defined by the stochastic process $B=\left\{B\left(t\right),t\geq 0\right\}$, as shown in Figure 5.

In this CTMC, the state $B_{i}$ represents the *i*-th charge interval, $\tau_{i}$ can be considered as the sojourn time into the state $B_{i}$ and, as a consequence, the transition rate between states $B_{i}$ and $B_{i+1}$ has to be set to λbti=1λbti=1τiτi. The discharge rates $\lambda_{bti}$ would be analogous to a set of battery failure rates, since these rates imply that the system will eventually reach a failed state. $B_{n}$ is an absorbing state that represents the $c_{\min}$ level. The probability of the battery being discharged ($c\left(t\right)\leq c_{\min}$) is $Prob\left\{B_{n}\left(t\right)\right\}$ and, consequently, the probability of the battery being a working ($c\left(t\right)\geq c_{\min}$) can be computed as $1-Prob\left\{B_{n}\left(t\right)\right\}$.

**Figure 6:**
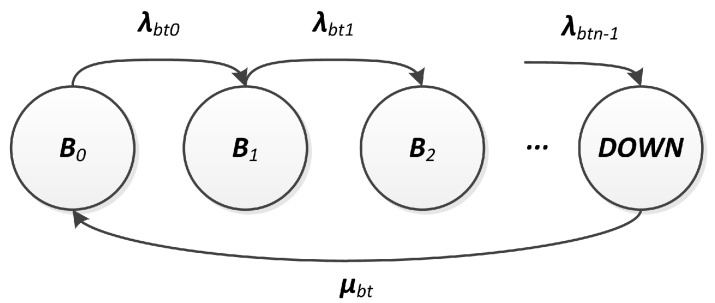
Battery model.

When the battery discharges below the minimal operational level ($c_{\min}$), it can be replaced or recharged, which is analogous to a repair. For the same reasons presented in Section 3.2.1, we model battery repair actions by a constant repair rate $\mu_{bt}$. For the battery availability evaluation, we propose the CTMC model presented in Figure 6, where the state *DOWN*is equivalent to the state $B_{n}$ from Figure 5, and it is the only one which triggers the repair action.

However, when the WVSN active–sleep cycle operation is considered, this approach becomes inaccurate [61]. For this purpose, considering that the battery current is almost constant in the active state and that it is negligible (≈0) in the sleep state, Costa et al. [14] characterize the active-sleep cycle by a duty-cycle DC, which is the percentage of time that a node stays in the active state. In this case, the amount of battery discharge in a interval $\tau_{i}$ is now proportional to DC. This is equivalent to assuming that the sojourn time for each state $B_{i}$ is τiτiDCDC. Therefore, the transition rate $\lambda_{bti}$ for each state can be redefined as follows:

(6) $$\lambda_{bti}=\frac{DC}{\tau_{i}}.$$

Analogous to the hardware modeling, the availability of the battery $bt=A_{bt}\left(t\right)$ is computed as the complementary probability of being on state DOWN.

### 3.4. Link Modeling

The link model consists of a description of the communication behavior between two nodes. Due to its wireless nature, we consider that link failures are transient. In addition, as a link is an abstract concept, we cannot materialize its repair. Thus, this repair action can be understood as the natural reestablishment of normal communication conditions after a failure, without a deliberate intervention. We assume that link failures also occur according to a Poisson process, leading to a constant failure rate $\lambda_{lk}$. Repairs are modeled analogously to the hardware case, assuming a constant repair rate, $\mu_{lk}$.

**Figure 7:**
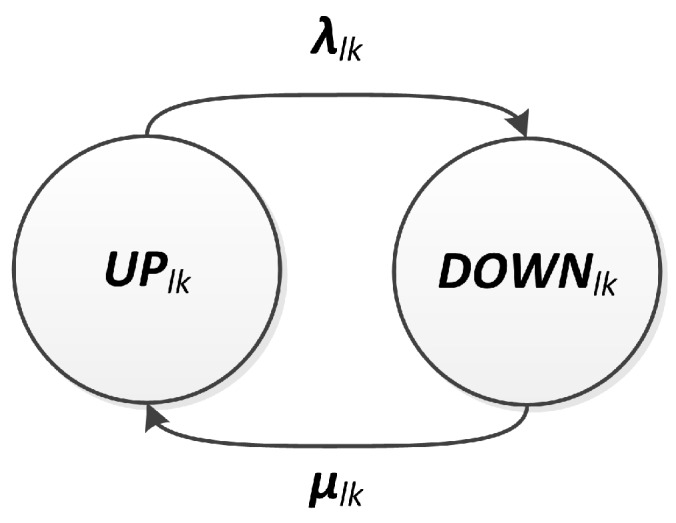
Link model.

That way, the link behavior with respect to availability is described as a binary relationship, which can be operable (UP) or failed (DOWN). In the former case, the link is operational, and in the latter it is failed. This behavior can be represented by a CTMC with two states ($UP_{lk}$ and $DOWN_{lk}$). Transitions between these two states are described by the failure and repair rates, $\lambda_{lk}$ and $\mu_{lk}$, respectively, as presented in Figure 7. Under the stated assumptions, the link availability, $lk=A_{lk}\left(t\right)$, can be computed as the probability of being on state $UP_{lk}$.

Link dependability is also related to the routing protocol. A link failure can change the network topological arrangement, excluding a path to the sink node, disallowing or delaying the delivery of part of the network visual information. Searching a new path to the sink depends directly on the used routing strategy. In order to consider this behavior in the dependability evaluation, routing protocols are an important issue, which needs to be properly evaluated.

Since there are too many routing protocols, it is very difficult to model all of them or even to find a general pattern. On the other hand, it would be very restrictive to model a specific protocol. Instead, some authors discuss and describe routing strategies that are common to several protocols. The most used strategies that can be found are DIRECT, FLOODING and HIERARCHICAL  [40,41,62]. In this paper, we address DIRECT and FLOODING strategies, and consider HIERARCHICAL as a future work.

The DIRECT strategy guarantees direct connection between each node and the sink node through one single hop. Although ideal for small networks, it may lead to high energy consumption, since it requires that radios to be set with high transmission power on nodes that are far away from the sink [40,63]. This routing strategy will be used for comparison purposes. In this paper, we consider the radio connectivity modeled by disk graphs, where each pair of nodes within a given distance threshold ρ are connected and can communicate directly with each other by a link [62].

The FLOODING strategy is a multi-hop strategy to discover multiple paths to the sink. Each node broadcasts a message to all of its neighbors, who repeat this procedure until the message is delivered to the sink or it is dropped out due to a maximum number of hops. This routing strategy is easy to implement but has some problems: duplicate messages and network overheads [62,63]. A good advantage of FLOODING is that it can deal with the loss of an intermediate node in a path, searching for new paths to the sink. This multiple path feature naturally provides a higher reliability, since the probability of a successful delivery of messages to the sink is higher with a high number of possible paths. In addition, as the nodes primarily communicate with their closest neighbors instead of communicating directly to the sink (which is probably more distant), each node can decrease the radio power to reach just the nodes in their vicinity, i.e., with a smaller distance threshold ρ. This implies a smaller power consumption, a slower battery discharging and therefore a higher reliability.

**Figure 8:**
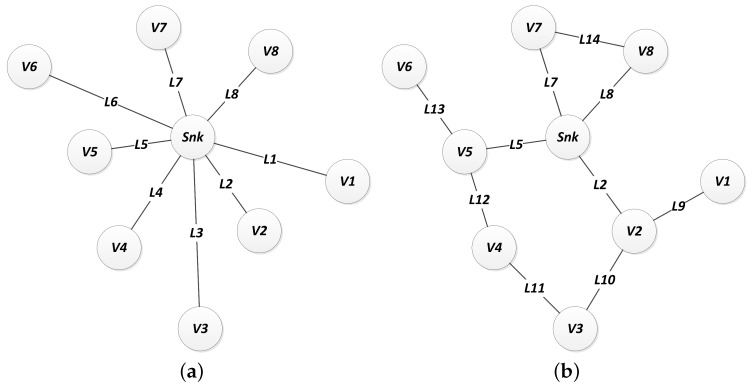
Routing strategies: (**a**) direct and (**b**) flooding.

Figure 8 illustrates the network topology of the same arrangement of nodes, managed by different routing strategies.

### 3.5. Integration of Models

In this section, we present how to integrate hardware, battery and link models (with the strategies of routing protocols) in order to obtain a single system model.

As aforementioned, the WVSN scenario considered in this paper and presented in Figure 1 is affected by different classes of failures. Coverage failures are the most important in a WVSN, and they are related to the inability of essential visual nodes to deliver their information to the sink. An essential node is one that directly participates on the required monitoring, so it determines the network failure condition (NFC). The NFC of a WVSN application consists of a logical expression that represents the combinations of components that, if failed, lead to the application failure [6]. Since we are considering WVSNs for area coverage, these combinations must be composed by essential visual nodes. Without the visual information from these nodes, the visual information gathered by the rest of the network is not enough to compose the required minimum area to be monitored [14].

For example, analyzing Figure 1, it is possible to notice that node V1 monitors a small area (rectangle) and the majority of this area is also monitored by nodes V2 and V3. Node V2 monitors a larger area than V1, but also with some overlapping related to V1 and V3. That way, the NFC for this network should probably be NFC=V2∨V3, depending on the application requirements. This failure condition is assessed *true* if V2 is assessed *true* (if the hardware of V2 fails) or if V3 is assessed *true* (if the hardware of V3 fails), indicating that the nodes V2 and V3 are essentials for visual monitoring. In this case, if at least one of these nodes fails, then the remaining visual nodes are not able to collect enough visual information to meet the application requirements.

On the other hand, the application could require a minimum area slightly larger than the coverage area of any visual sensor, it being necessary that at least two visual nodes are able to deliver their visual information to the sink in order to fulfill the application requirements. In other words, if any combination of two visual nodes fail, the application will fail, which implies NFC=(V1∧V2)∨(V1∧V3)∨(V2∧V3).

However, visual information of an essential node may also not reach the sink due to communication failures. For instance, if the node S2 fails, then there will be no way to deliver the visual information from both nodes V1 and V2. In the same way, if the link connecting nodes S1 and S2 fails, or if the link connecting nodes V2 and S2 fails, then there will be no way to deliver the visual information from both nodes V1 and V2, respectively. Those elements must appear in the evaluation of the whole system since they indirectly affect its dependability.

To cope with these issues, we model the system using Fault Trees (FT), similarly to Silva et al. [6] and Costa et al. [14], considering the previous classes of failures. A Fault Tree is a graphical procedure used to describe the combination of events that leads to a TOP event, in a treelike structure composed by events and logic gates [6]. In this case, the logic gates are used to represent cause–effect relationships among events, in an equivalent sense of the NFC. That way, the NFC is defined by a logical expression, composed by AND and OR gates, and has to be evaluated as *true* to identify an application failure.

**Figure 9:**
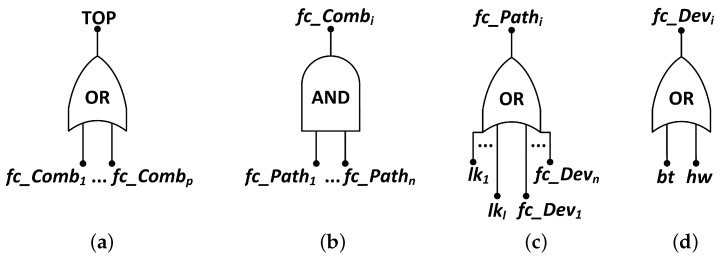
Fault Tree models of (**a**) network; (**b**) combinations; (**c**) paths; and (**d**) devices.

An AND gate indicates that a failure condition occurs if, and only if, all input events has occurred. In the OR gate case, the failure condition occurs if at least one input event has occurred [6,56]. The inputs of these gates are either single events or combinations of events which result from the output of other gates. The events at the bottom of the tree are referred as basic events and must be assigned to battery, hardware and link dependability functions (reliability or availability functions, according to the evaluation interest), resulting from the respective CTMCs’ evaluation. Figure 9 shows the logical gates configuration for each failure condition in order to represent the events’ dependency in an FT structure.

Figure 9a indicates that the application fails (the TOP event is assessed equals to *true*) if one or more given combination of essential nodes ($fc\_Comb_{i}$) fail. According to Figure 9b, a combination of nodes fails if all paths ($fc\_Path_{i}$) that connect the sink node to each node in the combination fail. As presented in Figure 9c, a path fails if any link ($lk_{i}$) or device ($fc\_Dev_{i}$) in the path from the device to sink node fails. Finally, a device (sensor node) fails if its hardware (hw) fails or if its battery discharges (bt), as shown in Figure 9d.

System dependability evaluation is considered to be, in the last stage, a Fault Tree analysis, which requires the evaluation of how the basic events (availability functions) associated with each network elements interact between them. In this context, the next section presents the methodology to automate the modeling process and to integrate the dependability evaluation with the SHARPE tool.

## 4. Proposed Methodology

**Figure 10:**
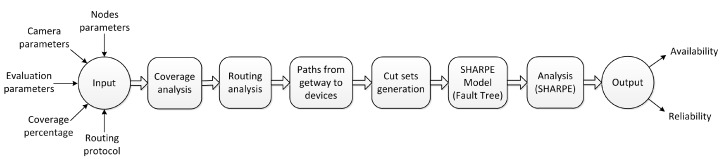
Overview of the methodology for dependability evaluation.

The proposed methodology takes advantage of the SHARPE tool to support hierarchical models. These models are structures that provide an overall model solution by composing individual model results, thus avoiding a large overall state space [13,64]. That way, it is possible to model at a higher level the dependency of events using Fault Trees, while at a lower level the complex behavior can be described by state-based models (Markov Chains, for instance). These formalisms are capable of modeling the required behavior and supporting the extraction of all the required metrics, like availability and reliability [65]. Figure 10 presents an overview of the proposed methodology, which is detailed in the following subsections.

### 4.1. Data Iinput

The automated framework developed to implement the proposed methodology requires some supplementary data from the user in order to characterize the network and the application requirements. This information is related to network configuration, visual coverage attributes, nodes and the evaluation process itself, which are:

1. Nodes parameters:
  - Number of visual nodes, nVS;
  - Number of scalar nodes, nSS;
  - (x,y) position of nodes, $\left(A_{x_{i}},A_{y_{i}}\right)$;
  - Radio communication range, $R_{c}$;
  - Hardware failure and repair rates, $\lambda_{hw}$, $\mu_{hw}$;
  - Battery discharge and repair rates, $\lambda_{bt_{i}}$ and $\mu_{bt}$, and number of stages, nStages;
  - Link failure and repair rates, $\lambda_{lk}$, $\mu_{lk}$;
2. Cameras parameters:
  - Viewing angle, θ;
  - Orientation, α;
  - Sensing radius, $R_{s}$;
3. Evaluation parameters:
  - Evaluation period, *T*;
  - Time step, $t_{s}$.
4. Minimum visual coverage percentage, $A_{min}$;
5. Routing protocol strategy, DIRECT or FLOODING.

### 4.2. Coverage Analysis

The first step aims to establish which visual sensor combinations are capable of monitoring the interest area A, in order to provide the minimum viewing area required by the application, $A_{min}$. This information will be used to compose the NFC: whenever none of these combinations can be formed by working nodes, the application will fail. This procedure is described in Algorithm 1, where the coverage area (*ca*) of each combination of sensors is computed and tested in Lines 2 and 3, respectively.  

**Algorithm 1:** NFC**Data**: nfc = NFC($VA_{min}$, VisualSensors[]); **Input**: Minimum Viewing Area, List of Visual Sensors and its parameters. **Output**: Network Failure Condition. 
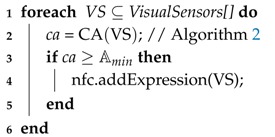


The coverage area computing routine is described in Algorithm 2, based on Definition 1, and is used in Line 4 of this algorithm. In that same line, the routine to compute overlapped areas (***oArea***) of covered regions is invoked. This routine is described in Algorithm 3 and it consists of the identification of the polygon formed by the vertices of overlapped regions. After that, the polygon area is computed according to Equation (7), based on the Shoelace equation [35,66], where |V| is the number of vertices of the polygon and $Vx_{i}$ and $Vy_{i}$ are its (x,y) coordinates. Those vertices must be in a clockwise or anti-clockwise order, since it is a requirement for the Shoelace algorithm.

**Algorithm 2:** Coverage area**Data**: *ca* = CA(VS, mArea); **Input**: List of sensor, monitoring area parameters. **Output**: Coverage area.

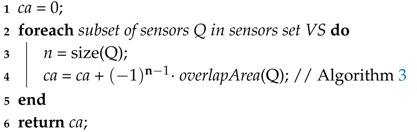


**Algorithm 3:** Overlap area**Data**: oArea = overlapArea(Q); **Input**: List of parameters of a subset of sensors. **Output**: Overlap Area. 
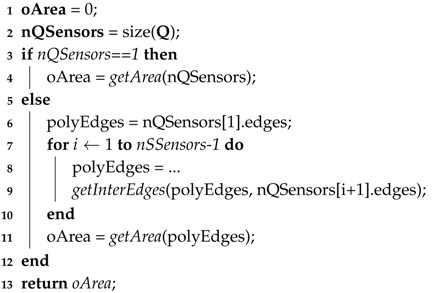

(7) $$oArea=\frac{\left|Vx_{\left|V\right|}.Vy_{1}-Vx_{1}.Vy_{\left|V\right|}+\sum_{i=1}^{\left|V\right|-1}\left(Vx_{i+1}.Vy_{i}-Vx_{i}.Vy_{i+1}\right)\right|}{2}$$

### 4.3. Routing Analysis

Admitting that visual nodes are able to monitor the minimum coverage area, the resulting visual information must reach the sink. This task is managed by the protocol, which imposes a set of communication rules in order to optimize aspects like power consumption, overhead, throughput and delivered messages. This way, the routing analysis defines the possible connections between nodes based on the selected strategy of routing protocol and nodes position.

These connections are mapped into an adjacency matrix Adj, which consists of a square matrix that represents an abstraction of the network topology. Each position $Adj_{ij}$ of the matrix represents the binary relation between the nodes associated to that position. That way, if there is a link connection between nodes *i* and *j*, then $Adj_{ij}=1$, otherwise $Adj_{ij}=0$. It is important to notice that $Adj_{ij}=Adj_{ji}$, ∀i,j. Figure 8 shows the network arrangement for different routing strategies. Notice that a different selection of routing strategy preserves the same node positions but generates different topological arrangements, which leads to a different adjacency matrix.

Algorithm 4 details how to proceed with the routing analysis, starting by creating the adjacency matrix, based on an identity matrix with dimension equal to the number of nodes (Line 2). This means that each node is connected with itself. Then, the adjacency matrix will be updated according to the selected routing strategy. If the selected strategy is DIRECT, a connection between each sensor node and the sink will be created, as shown in Line 7. For that, it is supposed that each node has enough radio transmission power to directly communicate to the sink using the DIRECT strategy. On the other hand, if the selected strategy is the FLOODING, a connection between two sensor nodes *i* and *j* will be created (Line 14) if the distance between them is less than or equal to their radio communication range, i.e, if $d\left(i,j\right)\leq R_{c}$ (Line 13). In this case, it is important to remember that a given node will probably communicate with the sink through a sequence of message re-transmissions. Therefore, it can be considered a smaller radio communication range, which implies that each node can reduce its radio power, generating a smaller power consumption and a slower battery discharging.   

**Algorithm 4:** Routing**Data**: Adj = Routing(strategy, $R_{c}$, nodes[]); **Input**: Strategy of routing protocol, Radio range, List of all nodes and its parameters. **Output**: Adjacency matrix of network. 
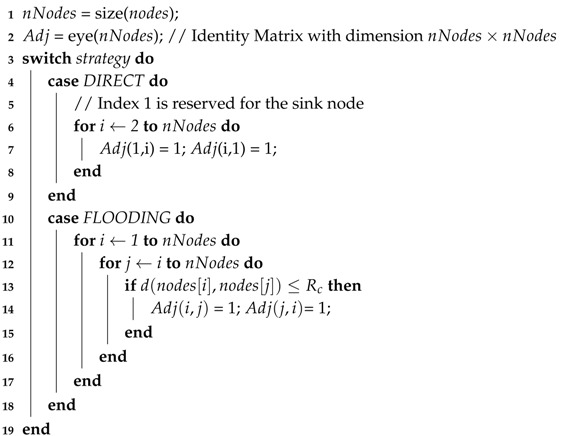


### 4.4. Paths and Cut Sets Generation

Using the network topology described by adjacency matrix, the next step is to discover which nodes and links are involved in the communication between the sink and the nodes that perform the successful area monitoring, i.e, which nodes and links are responsible to route the information from the nodes belonging to the NFC to the sink. For this task, a depth-first search (DFS) in the adjacency matrix starting from the leaves (nodes in the NFC) is performed, until finding the root (sink).

Algorithm 5 creates a set of paths from each node belonging to the NFC. This is performed by the invocation of Algorithm 6 (Line 5), which recursively goes through the adjacency matrix to find new neighbor nodes (Line 9) and adding these nodes and its links (Lines 11 and 12) until finding the sink (Line 4). In order to avoid cycles, nodes that have already been selected for the path are ignored (Line 10).

Each found path is called a *cut set*. In a Fault Tree analysis, a cut set is a subset of events whose simultaneous occurrence leads to the occurrence of the TOP event. Some authors go further [6] and find the *minimal cut set*, which is a cut set that does not contain any other cut set. A minimal cut set is important to reduce the number of mathematical operations required to compute the TOP event, which can be significant in a large FT. On the other hand, since the available computational tools already cope with this issue, this task is ignored in this paper.    

**Algorithm 5:** Paths Discovery**Data**: paths = PathsDiscovery(Adj[][], NFC); **Input**: Adjacency Matrix, Network Failure Condition. **Output**: Set of All Paths from nodes that interfere on NFC. 
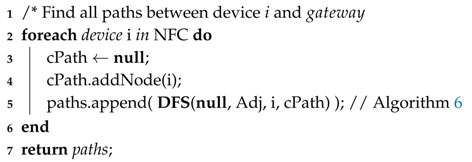


**Algorithm 6:** DFS**Data**: paths = DFS(paths, Adj, cDevice, cPath); **Input**: Collection of Paths, Adjacency Matrix, Device to be inserted, Current Path. **Output**: New Collection of Paths. 
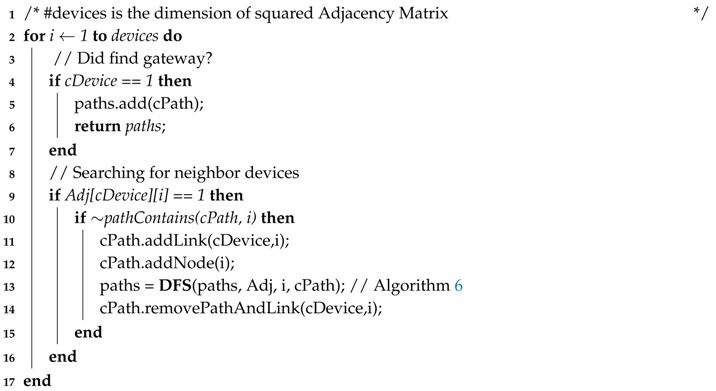


### 4.5. Fault Tree Model Generation

Following the methodology flow, the model for the whole system must be generated according to the SHARPE language and syntax. Algorithm 7 looks into each path found in the previous step and, for each network element, the algorithm writes the corresponding SHARPE code to a text document that will be the input of SHARPE. This code describes the structure of the Fault Tree and the CTMC models. According to Figure 9, this structure is an OR-gate per device including battery and hardware events as inputs (Line 8), and an OR-gate per path, including device and link events as inputs (Line 13). Then, the structure is completed by a AND-gate including each path event as input (Line 15). Finally, the generated code is organized in a text document, getting each reference between hardware, battery and link events and generating their CTMC code (Line 17). The availability events of hardware and links, according to Figure 4 and Figure 7, and availability events of battery, according to Figure 6, are associated with their CTMCs in Lines 6, 11 and 7, respectively. Each availability event is called a basic event. If a basic event occurs two or more times in an FT, it is called a repeated event. Notice that, in order to use this methodology to perform a system reliability evaluation, it is only necessary to remove the repair activities from the CTMC models, which means setting the repair rates $\mu_{hw}$, $\mu_{bt}$ and $\mu_{lk}$ equal to zero.    

**Algorithm 7:** Fault Tree Generation**Data**: FaultTree = FaultTreeGeneration($\lambda_{hw}$, $\mu_{hw}$, $\lambda_{bti}$, $\mu_{bt}$, $\lambda_{lk}$, $\mu_{lk}$, btStages, Adj[][], NFC); **Input**: Failure and Repair Rates of Hardware, Battery and Link; Battery Stages; Adjacency Matrix, Network Failure Condition. **Output**: Fault Tree associated to the Network Failure Condition. 
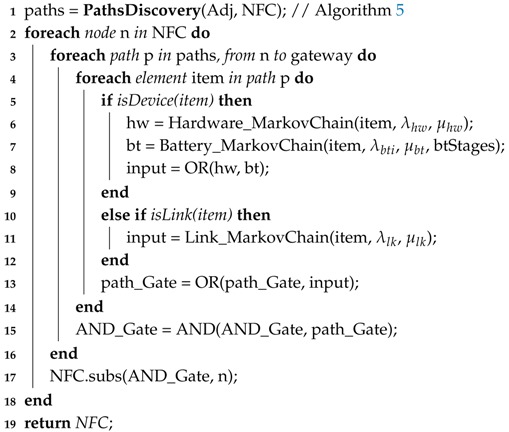


### 4.6. Fault Tree Analysis and Data Output

The Fault Tree analysis is totally performed by the SHARPE tool, facilitating the time-consuming tasks of describing and evaluating large and complex systems. SHARPE receives a text document with the Fault Tree description, the related CTMCs and the dependability attributes to be assessed for the evaluation period *T*, considering the discrete time step, $t_{s}$. First, the SHARPE tool evaluates the individual CTMC models of hardware, battery and link and obtains their respective availability function. These functions are then used as input events of Fault Tree gates, according to the described model. At the end, SHARPE evaluates the FT and returns another text document with the values of the dependability metrics (e.g., availability) for each instant of time. The integration with the SHARPE is fully automated by the developed framework.

The source code of the algorithms presented are available in [67].

## 5. Results and Discussion

In this section, we present some results obtained when using the proposed methodology to evaluate WVSNs’ dependability. That way, some examples are given showing different types of analysis allowed by the proposed methodology. For all examples, it is assumed that each device uses the same model of battery, which is an alkaline battery (AA/R6) with the following specifications:

- $c_{0}$ = 3000 mAh;
- *H* = 25 h;
- *I* = 100 mA;
- η = 1.3;
- DC = 50%.

For this case, it would take 25 h to discharge the battery considering an average operation current of 100 mA. Considering a 50% duty-cycle, this will increase the operating time up to 50 h. It is assumed that a node cannot work properly with a battery capacity lower than $c_{\min}=500$ mAh. Thus, the modeling of the battery by an approximation to a stochastic process is given as follows. Suppose that the battery discharges through n=4 stages, and then, according to Section 3.3, each stage discharges c0-cminc0-cminnn=625 mAh. This means that each stage duration $\tau_{i}=t_{i+1}-t_{i}$ (i=0,…,n-1) can be found solving Equation (5) to the values $c_{0}=c\left(t_{0}\right)=c\left(0\right)=3000$ mAh, $c_{1}=c\left(t_{1}\right)=2375$ mAh, $c_{2}=c\left(t_{2}\right)=1750$ mAh, $c_{3}=c\left(t_{3}\right)=1125$ mAh and $c_{4}=c\left(t_{4}\right)=500$ mAh. This implies in $\tau_{0}$ = 4.1235 h, $\tau_{1}$ = 6.0297 h, $\tau_{2}$ = 7.0465 h and $\tau_{3}$ = 7.8003 h. Thus, according to Equation (6), $\lambda_{bt0}$ = 0.1213/h, $\lambda_{bt1}$ = 0.0829/h, $\lambda_{bt2}$ = 0.0710/h and $\lambda_{bt3}$ = 0.0641/h. Once discharged, we consider that it takes 2 h to repair (replace or recharge) the battery, so $\mu_{bt}$ = 1/2 = 0.5/h. It is important to remark that increasing the number of battery stages results in a better approximation to the real battery discharging behavior [60].

With respect to the visual nodes and theirs parameters, we assume a viewing angle θ = $60^{\circ }$ and a sensing radius $R_{s}$ = 150 m for all considered visual sensors. In addition, we consider that the radio communication range is $R_{c}$ = 180 m. These device parameters are used for the communication scenarios of Examples (Section 5.1 and Section 5.2).

### 5.1. Example 1

**Figure 11:**
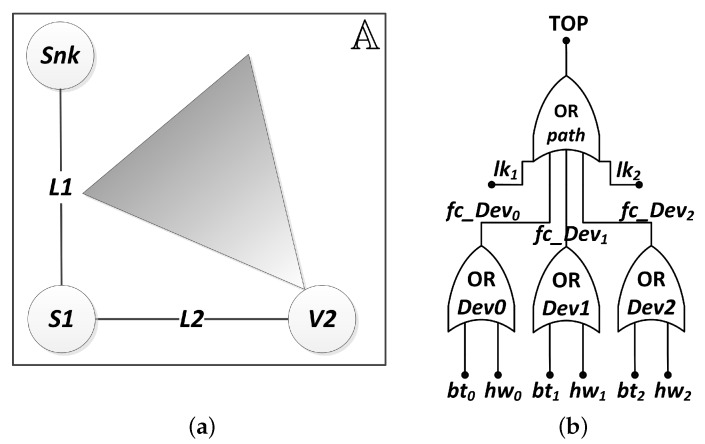
(**a**) system topology and (**b**) its Fault Tree (FT).

In order to highlight the proposed approach, first we evaluate the dependability of a small network, composed by a sink node (Snk), a scalar node (S1) and a visual node (V2), as shown in Figure 11a. The visual node is represented by a circle attached to a triangle, which is the camera’s FoV.

The system uses FLOODING strategy and considers the occurrence of 1 hardware failure per year and one link failure per every two days, which implies hardware and link failure rates as $\lambda_{hw}$ = 1.1416 ×$10^{-4}$/h and $\lambda_{lk}$ = 0.02083/h, respectively. In addition, it is considered that it takes 72 h to repair the hardware and 15 min to a link to be reestablished, which implies the hardware and link repair rates as $\mu_{hw}$ = 0.013894/h and $\mu_{lk}$ = 4/h, respectively. It is important to notice that the assignment of values to link failure and repair rates depends on several aspects, e.g., network physical layer, communication environment, network deployment, etc. However, since the goal of the paper is to present a methodology to evaluate and compare different network implementations, the method to define these rates is assumed as secondary in this paper.

Since we have only one visual node, Algorithm 1 identifies node V2 as the unique one responsible for the monitoring. Therefore, the network failure condition must be NFC = V2, i.e., if the node V2 fails, the visual application fails. Then, the network topology is known by the Algorithm 4, and it is represented by the following adjacency matrix:

(8) $$mAdj=\left[\begin{array}{ccc} 1 & 1 & 0\\ 1 & 1 & 1\\ 0 & 1 & 1 \end{array}\right].$$

This means that the sink (index 1 of the matrix) is connected only to the scalar node (index 2 of the matrix), whereas the scalar node is also connected to the visual node (index 3 of the matrix). This information is used by Algorithm 5 to find all paths from the NFC devices (V2, in this case) to the sink, by a depth-first search (Algorithm 6). There is only one path with this feature, which is V2→L2→S1→L1→Snk, where L1 is the link connecting Snk and S1, and L2 is the link connecting S1 and V2. Thus, Algorithm 7 will generate a Fault Tree that represents this behavior, as shown in Figure 11b. Notice that the logic expression indicates that if any link ($lk_{1}$, $lk_{2}$) or device ($fc\_Dev_{0}$, $fc\_Dev_{1}$, $fc\_Dev_{2}$) fails, the path will be interrupted and the application fails. Additionally, as this system has only one device associated with the NFC and one path from this device to the sink, then the AND-gate related to the failure condition of combinations (Figure 9b) has only one input, and the same happens to the OR-gate related to the failure condition of paths (Figure 9c). These gates were replaced by a bypass connection in the Fault Tree structure.

**Figure 12:**
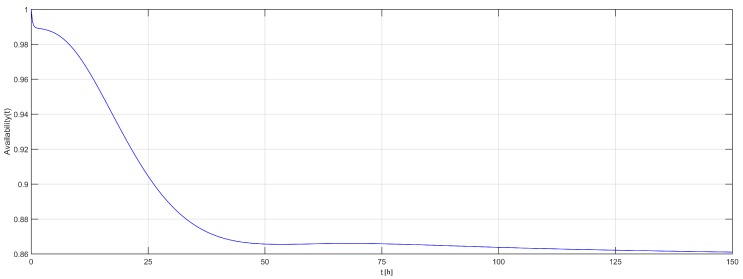
Graphical methodology output.

The generated Fault Tree (SHARPE) model can be seen in Listing 1. Notice that each event (Lines 2–9) is represented by an availability function. For instance, in Line 9, there is a reference to the availability of the Markov Chain model (BT_model_Dev0) of the sink’s battery (Dev0). This model is described in Listing 2. Finally, Listing 3 shows how to invoke the availability evaluation of the system. The evaluation period is *T* = 150 h, with a time step of $t_{s}$ = 15 min (0.25 h) (Line 2). All of these models have been generated by Algorithm 7. Figure 12 shows the graphical output, indicating that, after 50 h of execution, the availability of the system is slightly higher than 86%.

Listing 1: Fault Tree model.

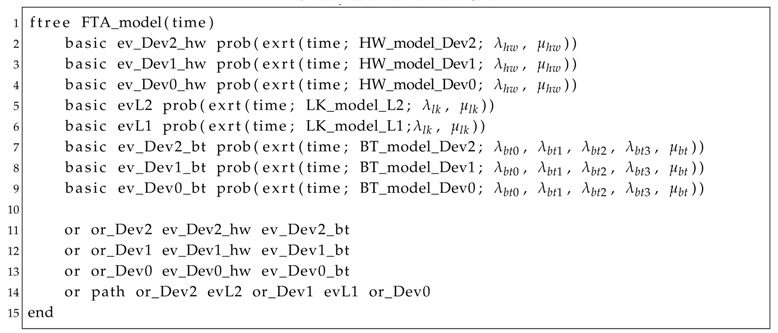



Listing 2: Markov Chain model.

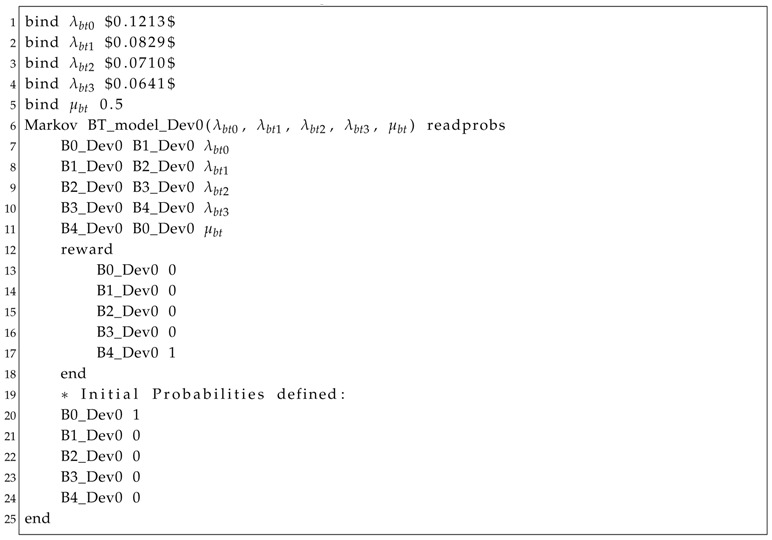



Listing 3: Availability evaluation.

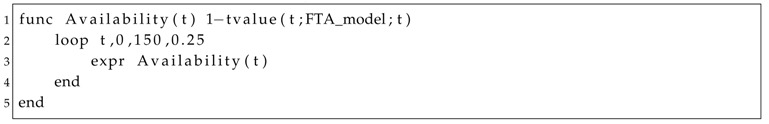



### 5.2. Example 2

**Figure 13:**
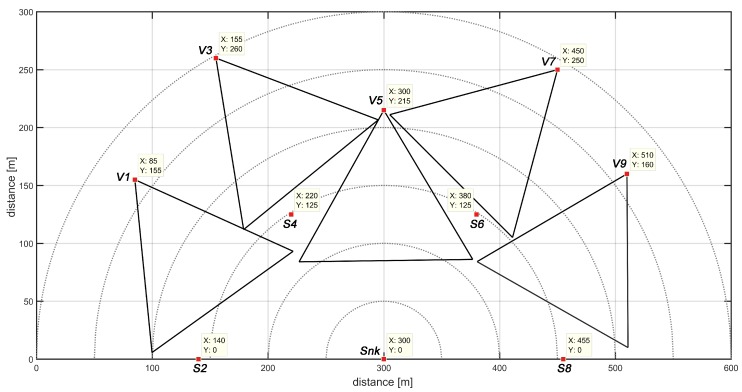
Area coverage in Example 2.

This example set up a larger system in order to analyze the effects of routing protocols strategies, links and battery discharges upon the availability evaluation. In addition, the availability with respect to parameters’ variation (failure and repair rates) is analyzed. For this purpose, we consider the WVSN represented in Figure 13, with five visual nodes, four scalar nodes and one sink node. The monitored area has $18\times 10^{4}$ m${}^{2}$ and the minimum required coverage area is 20%, which is $A_{min}=36\times 10^{3}$ m${}^{2}$. This implies the following network failure condition:

(9) $$\begin{array}{c} \mathrm{NFC}\;=\;(V1\wedge V3)\;\vee \;(V1\wedge V5)\;\vee \;(V1\wedge V7)\;\vee \;(V1\wedge V9)\;\vee \;(V3\wedge V5)\;\vee \\ \;\vee \;(V3\wedge V7)\;\vee \;(V3\wedge V9)\;\vee \;(V5\wedge V7)\;\vee \;(V5\wedge V9)\;\vee \;(V7\wedge V9), \end{array}$$

where operator “∧” indicates an AND gate and operator “∨” indicates an OR gate. The hardware and link failure rates are $\lambda_{hw}$ = $1.1416\times 10^{4}$/h and $\lambda_{lk}$ = 0.0417/h, respectively, which means one hardware failure per year and one link failure per day, and hardware and link repair rates of $\mu_{hw}$ = 0.0208/h and $\mu_{lk}$ = 2/h, respectively, which means one day to repair the hardware and 30 min to reestablish a link.

**Figure 14:**
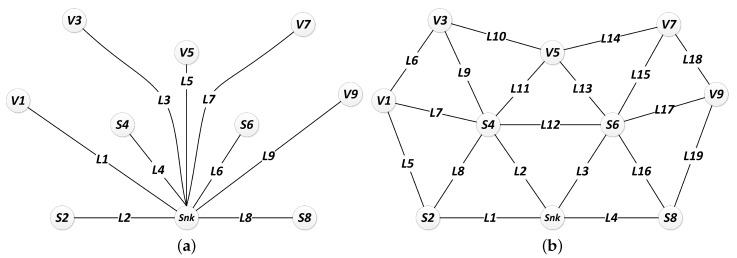
Network topology of routing strategies (**a**) DIRECT and (**b**) FLOODING.

**Figure 15:**
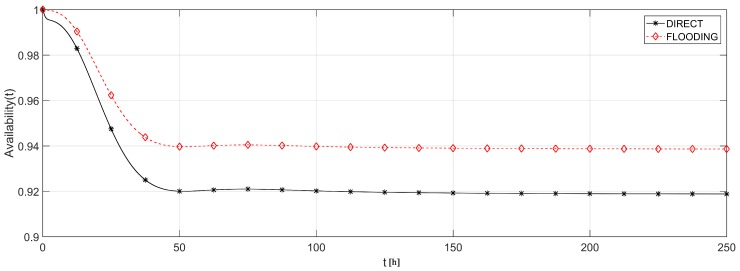
Availability of the network of Figure 13.

First, we analyzed the effect of the routing protocols’ strategies. Figure 14 shows the network topology for DIRECT and FLOODING strategies. Figure 15 shows the availability evaluation of those topologies. As it was expected, the FLOODING strategy presents higher availability (≈94%) then the DIRECT strategy (≈92%) due to the fact that there are multiple possibilities to reach the sink. This is an interesting result because, besides providing a higher availability, FLOODING strategy also allows each visual node to reduce its radio transmission power and therefore to save battery. However, this methodology is not able to measure such power consumption savings yet. For this purpose, the battery modeling should be directly integrated into the routing protocol, in order to associate the average discharging current according to the selected routing strategy. This task could be performed by simulation, which is out of the scope of this work.

**Figure 16:**
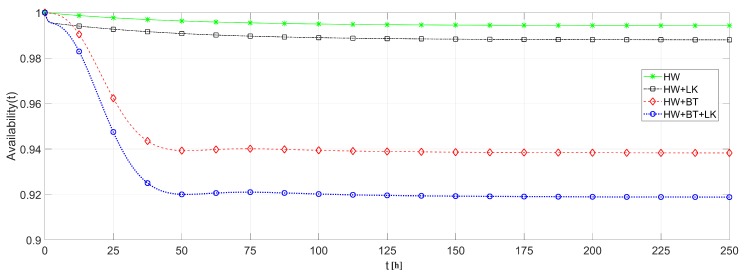
Availability of network of Figure 13: effects of battery and link failures with DIRECT strategy.

**Figure 17:**
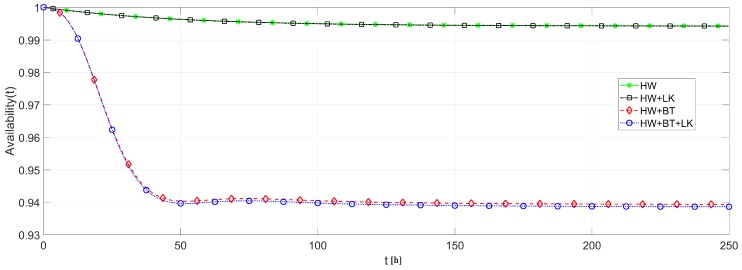
Availability of network of Figure 13: effects of battery and link failures with FLOODING strategy.

We also analyzed the effects of link and battery failures upon the system availability, using each of the routing strategies. To cope with this analysis, the system availability is evaluated in four different ways: (i) considering only hardware failures to provide a comparison basis; (ii) considering hardware and link failures to highlight just the link failure effects; (iii) considering hardware and battery failures to highlight just the battery failure effects; and (iv) considering hardware, link and battery failures together to obtain the overall system behavior. Figure 16 plots the result of these analysis based on DIRECT strategy and Figure 17 based on FLOODING strategy.

From Figure 16 and Figure 17, it is possible to notice that the battery discharge is the component that causes a major effect upon the system availability, since the battery discharges faster than the other failures’ occurrences. On the other hand, link failures have a smaller impact due to their transient behavior, which strongly depends on the routing protocol. Notice that link failures barely interfere in a network with FLOODING strategy, while it presents a considerable effect upon a network with DIRECT strategy. This is due to the fact that, in a network with FLOODING strategy, a lost path is quickly replaced by another one.

### 5.3. Example 3

**Figure 18:**
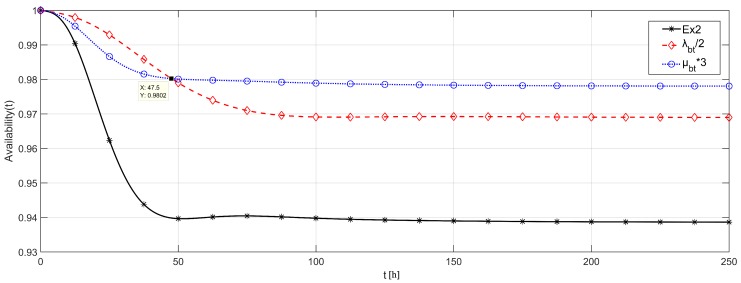
Availability evaluation for system design.

Finally, we presented an example of how to use the proposed methodology to guide the system design steps. In this case, we considered the network of Example 2, assuming that the cost of duplicating the quality of the battery is similar to the cost of reducing three times its repair time. This assumption means that the cost of buying a higher capacity battery is similar to buying a faster battery charger. In that case, the new battery would present failure rates with half of the value from Example 2 or repair rates three times higher. Thus, which is the better decision? Figure 18 provides information resulting from that analysis, comparing the availability of the system from Example 2 with the availability of the system with either the new battery, or the new repair approach. We considered similar failure and repair rates from Example 2, with FLOODING strategy.

Both changes imply a significant improvement of the system availability, where, during the first 47.5 h, the new battery provides a higher availability, while the new battery repair approach provides a higher asymptotic availability. If it is required that the application runs for less than two days, then it is preferable to invest in a better battery. Otherwise, a faster battery repair will allow the application to be available for more time.

## 6. Conclusions

In this paper, we proposed an automated methodology to analytically evaluate the dependability of Wireless Visual Sensor Networks, considering coverage, hardware, battery and link failures, and the impact of different routing protocols strategies upon the network communication behavior. The proposed methodology is implemented by an automated framework integrated with the SHARPE tool, which takes advantage of SHARPE’s support of hierarchical models. The used algorithms were clearly described and some examples of how to use the methodology were also presented, including how to use the proposed methodology to guide important project decision making.

Actually, there are still some relevant topics to be addressed. A more comprehensive type of failures should be considered, such as common-cause failures (CCF) and specific coverage failures as occlusion, as well as other routing protocol strategies, such as hierarchical protocols. Nevertheless, the considered set of failure types forms a coherent set that can be easily applied to multiple communication scenarios. This methodology could be also used together with a simulation approach, in order to provide more accurate evaluations, namely to estimate communication failure and repair rates. Similar investigations could also be performed for targets coverage and considering different coverage relevancy, where the application dependability will be more dependent on monitoring of critical areas or targets. Such remarks will be considered in future works.
